# Recent Developments in Metal-Based Drugs and Chelating Agents for Neurodegenerative Diseases Treatments

**DOI:** 10.3390/ijms20081829

**Published:** 2019-04-12

**Authors:** Thais A. Sales, Ingrid G. Prandi, Alexandre A. de Castro, Daniel H. S. Leal, Elaine F. F. da Cunha, Kamil Kuca, Teodorico C. Ramalho

**Affiliations:** 1 Laboratory of Molecular Modeling, Department of Chemistry, Federal University of Lavras, 37200-000 Lavras, MG, Brazil; thaissales194@hotmail.com (T.A.S.); ingrid.prandi@gmail.com (I.G.P.); alexandre.a.castro@hotmail.com (A.A.d.C.); elaineffdacunha@gmail.com (E.F.F.d.C.); 2 Department of Health Sciences, Federal University of Espírito Santo, 29932-540 São Mateus, ES, Brazil; daniel.leal@ufes.br; 3 Department of Chemistry, Faculty of Science, University of Hradec Kralove, 500 03 Hradec Kralove, Czech Republic; 4 Biomedical Research Center, University Hospital Hradec Kralove, 500 03 Hradec Kralove, Czech Republic

**Keywords:** metallodrugs, chelating agents, drug development

## Abstract

The brain has a unique biological complexity and is responsible for important functions in the human body, such as the command of cognitive and motor functions. Disruptive disorders that affect this organ, e.g., neurodegenerative diseases (NDDs), can lead to permanent damage, impairing the patients’ quality of life and even causing death. In spite of their clinical diversity, these NDDs share common characteristics, such as the accumulation of specific proteins in the cells, the compromise of the metal ion homeostasis in the brain, among others. Despite considerable advances in understanding the mechanisms of these diseases and advances in the development of treatments, these disorders remain uncured. Considering the diversity of mechanisms that act in NDDs, a wide range of compounds have been developed to act by different means. Thus, promising compounds with contrasting properties, such as chelating agents and metal-based drugs have been proposed to act on different molecular targets as well as to contribute to the same goal, which is the treatment of NDDs. This review seeks to discuss the different roles and recent developments of metal-based drugs, such as metal complexes and metal chelating agents as a proposal for the treatment of NDDs.

## 1. Introduction

Neurodegenerative diseases (NDDs) are characterized by progressive dysfunction and loss of neurons, which leads to distinct involvement of functional systems defining clinical presentation [1,2,3]. Among the pathologies that belong to this class, it can be highlighted Alzheimer’s disease (AD), which is the most common cause of dementia, along with frontotemporal dementia (FTD), amyotrophic lateral sclerosis (ALS), dementia with Lewy bodies (DLB), Parkinson’s disease (PD), Huntington’s disease (HD), Friedreich’s ataxia (FRDA) and prion disease [4,5]. These NDDs cause social and economic overload in societies worldwide. The speedy growth in knowledge, related to the pathogenic mechanisms and disease-associated biomarkers, has sped up the design of novel diagnostic tools and therapeutic techniques [6].

The neurodegenerative diseases onset take place mainly in people above the age of 45 years old [7,8]. According to the UN (United Nations) world population prospects, the number of individuals aged 60 or over on the planet is estimated to grow about four times over the next 30 years, predicting that diagnoses of dementia may also rise [9]. The symptoms progressively advance with the disease, reducing the capacity for independent living, and, at long last causing, death [10,11]. The course of the disease has an average duration of 10–15 years, regarding the onset of clinical symptoms, but presenting a significant variability amongst individuals [10,11]. Researches around the world strive to develop novel remediation techniques for NDDs, being a major concern due to the growing increase of the elderly population and increasing burden on patients, families, and society. However, the multifactorial nature of these NDDs diseases is one of the main obstacles to drug development [12]. Although significant advances have been made in the last decades to comprehend the underlying genetic and biological causes of these diseases, only some symptomatic treatments are available [9,13].

Over the years, a great diversity of novel compounds and molecular targets have been studied, aiming the development of new NDDs treatments. One of the most common targets, in this context, is metal homeostasis. It is widely known that the presence of metal ions is essential for living organisms, and many important enzymes require the presence of these ions to exert its catalytic activity. Among the 1371 known structure enzymes registered in the PDB SwissProt Enzyme classification database (PDBProtEC; PDB: Protein Data Bank), it is estimated that approximately 40.7% of them require a metal cofactor [14]. However, the accumulation of metals such as copper, iron and zinc in the brain is pointed out as the cause of oxidative damage, as well as other critical roles in the brain of patients suffering from NDDs [12,15,16]. Based on these shreds of evidence, several metal chelating agents are being developed with the purpose of regulating the concentration of metal ions in the brain. Among these, a new generation of weaker chelating agents, called metal protein attenuating compounds (MPACs), has the ability to only regulate the abnormal concentrations of the metal ions, without causing damage to other processes that require the presence of metals [17]. In addition to chelating drugs, it can be highlighted other new approaches to NDDs drug design, such as the development of multifunctional molecules, able to simultaneously combat several pathological features [12]. These compounds, including natural products, are able to play many roles, acting as chelating agents, antioxidants, anti-inflammatory drugs, peptide-aggregation reducers and AChE inhibitors, among others [18,19,20,21,22,23,24,25].

Curiously, there is another class of compounds, the metal-based drugs, with opposite biochemical activity to chelating agents, which is also being developed to act in the treatment of NDDs. Metallodrugs can act on a variety of molecular targets, and can, for example, be able to bind to amyloid-β species [26], or even to simulate the role of important enzymes, such as superoxide dismutases (SOD) [27]. Considering the wide range of studies that have been developed in these two different approaches, and for NDDs treatment in general, this review brings novel perspectives into the NDDs-related therapies, which treatment is dictated by the presence of metal-based or metal chelating drugs, playing quite important roles in the remediation process.

## 2. Neurodegenerative Diseases (NDDs)

NDDs are characterized by the progressive loss of neuronal function. There are many pathologies that belong to this class. As said before, the most ordinary cause of dementia is AD. Together with PD, FTD, ALS, DLB, HD, FRDA, and prion disease [4,5], these NDDs cause social and economic overload in societies worldwide.

The neurodegenerative disease onset takes place mainly in people above 45 years of age The major symptoms related to NDDs are dementia, cognitive decline, high order brain alterations, motor impairment, behavioural modification, psychosis and emotional disturbance [1,7]. The first symptom, dementia, is a common neurological disease of heterogeneous origin, whose most important risk factor is ageing. Dementia damages memory and other cognitive functions, interfering with the patient’s ability to maintain usual daily activities. According to the UN world population prospects, the number of individuals aged 60 or over is estimated to grow about four times over the next 30 years, bringing the prediction that diagnoses of dementia will also rise [9]. The severity of the symptoms progressively advances with the disease development, leading these patients to a reduced capacity for independent living, and, at long last causing, death [10,11]. The characteristic course of the disease has an average duration of 10–15 years, regarding the onset of clinical symptoms, but presenting a significant variability amongst individuals [10,11].

### 2.1. Alzheimer’s Disease

Alzheimer’s disease (AD) is a well-known form of dementia, affecting an increasing number of people around the world. Some available data indicate an exponential rise regarding the number of cases of AD, reinforcing the need to develop effective treatments and therapies [28,29]. AD is an illness of the central nervous system (CNS), being this frame of neurodegeneration irreversible to date. It progressively damages patient memory and cognition, commonly in the geriatric population [30,31]. The disorder is often classified based on the age of onset, for instance, early and late-onset AD. Early onset AD is responsible for about 1–6% of cases and manifests in individuals up to 60 years. On the other hand, late-onset form accounts for around 90% of all cases, presenting an age at onset later than 60 years [28,32].

In current days, AD is reported as being a chronic and progressive neurodegenerative process, characterized by the accumulation of amyloid-β protein (Aβ) in amyloid plaques and by the formation of neurofibrillary tangles resulting from the hyperphosphorylation of the tau protein associated with cellular microtubules [33,34]. Overaccumulation of these species fatally leads to synaptic dysfunctions, resulting in neuronal losses. Concerning AD, the disease-involved molecular mechanisms are not fully elucidated so far, but it is known that some risk factors favour the onset and worsening of the illness, such as advanced age. Some hypotheses strive to explain the factors underlying the pathological frame of AD. In this context, the main theories are related to the amyloid-β cascade hypothesis, oxidative hypothesis, tau protein hypothesis and cholinergic hypothesis [35,36,37].

The theory related to the Aβ fragments deposits suggests the formation of toxic soluble oligomers, which give rise to insoluble neuritic plaques [38]. It is also highlighted the appearance of inflammatory processes [39,40]. Recent indications show that this process plays a role in the AD pathogenesis, and its comprehension and control could be of significant importance in preventing or delaying the onset of CNS diseases [41,42]. The major hypotheses which surround Aβ generation and tau protein hyperphosphorylation are described in more details next.

#### 2.1.1. Amyloid-β Cascade Hypothesis

AD is well known by the damages caused in synaptic and neuronal functions, leading to a gradual loss of neurons, as previously commented [43]. The amyloid cascade hypothesis reports the role of Aβ fragments in the production of plaques and the formation of neurofibrillary tangles (NFTs) [44,45].

It is important to highlight that the Aβ peptide is generated from the amyloid precursor protein (APP), with aggregation and formation of senile plaques. APP is a membrane glycoprotein present in neurons, being a substrate for two different enzymes: α- and β-secretases. After the first APP cleavage catalysed by α- or β-secretase, a proteolysis process occurs, being assisted by a third enzyme known as γ-secretase [46]. The soluble peptide p3, which is the product formed from the cleavage by α- and γ-secretases, demonstrates no tendency to form aggregates, which is a characteristic of the proteolysis via the non-amyloidogenic pathway. In addition, the processing by β- and γ-secretases leads to the production of neurotoxic fragments of Aβ [47].

A range of works has suggested that soluble oligomers demonstrate higher toxicity than fibrils, and, because of this, AD is clearly related to the existence of Aβ oligomers and deposits within the brain [46]. This point of view is supported by indications that the concentration of senile plaques within the brain of some patients does not predict the level of dementia; furthermore, a reduction in their concentration, by employing effective therapeutic agents, does not reverse the pathological frame of the disorder [47,48].

**Figure 1:**
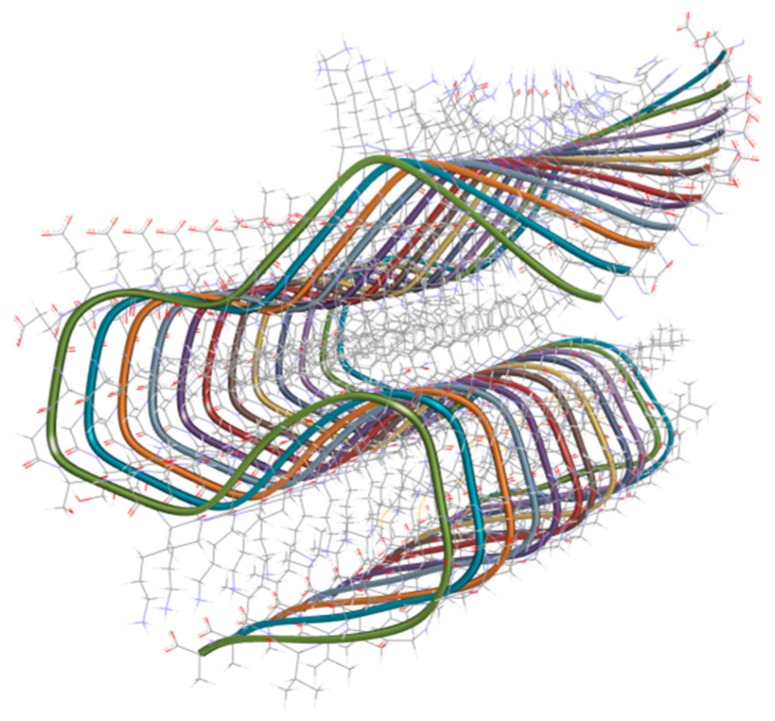
Three-dimensional ribbon structure of the 42-residue amyloid-β fibril (PDB code: 2MXU) [51].

Commonly, Aβ peptides are constituted by species with variable lengths, ranging from 37 to 42 residues. The fragment with 42 residues (Aβ_42_; Figure 1) presents pro-aggregating properties. A pathological increase in the production of Aβ_42_ may indicates the presence of AD [49]. In this context, appropriate tools for searching for bioactive agents against Aβ concern on the inhibition of Aβ aggregates formation, decreasing Aβ fibrils generation [47]. Hence, the development of effective therapies against Aβ is based on the reduction of the levels of highly toxic forms of Aβ, as well as insoluble fibrils [47,50].

Aβ could favour the appearance of an inflammatory process, which contributes to cognitive decline. AD risk factors could increase the possibility of acquiring dementia, and systemic inflammation. Among the factors that favour the onset of the disorder, it can be highlighted obesity, advanced age and even traumatic brain damage [41]. Remarkably, midlife obesity is demonstrated as being a risk factor in AD along with a sedentary lifestyle, high-cholesterol diet and decreased physical activity [41,52].

Nowadays, several novels β-secretase substrates have been identified [53,54]. Indications obtained from some trials have demonstrated that inhibitors of β- and γ-secretases could be effective in the treatment of early forms of AD. New works suggest that the formation of Aβ aggregates comes prior to the onset of the primary symptoms of dementia, capable of bringing about some improvement for the early pathogenic frame of AD [47,55]. The relationship between aggregates and soluble oligomers of Aβ and gradual phosphorylation of the tau protein is a necessary discussion to support theories referring to the development and evolution of AD [56].

Drug development for AD is an important step to provide a better life quality for patients with this illness. This field focuses on directly preventing the formation of Aβ_42_ or removing the existing Aβ_42_, along with the prevention of tau-related toxicity [57]. It is important to keep in mind that the Aβ cascade is followed by some neuropathological processes, including tau phosphorylation, agglomeration of paired helical filaments, astrocyte action, disrupted ion homeostasis, along with oxidative stress [58]. Currently, the available treatment forms provide only symptomatic benefits for AD people, leading to the necessity of developing more promising therapies [59].

The provision of high-quality assistance, services and information can be a potent tool, making the difference in the life of people diagnosed with AD [40,43]. Worryingly, diverse attempts which aim to develop more effective therapies have not so far reached success, with many high-profile clinical trials failing to demonstrate benefits. It is worth mentioning that part of this failure is probably due to the huge emphasis on the amyloid cascade as a molecular target for disease modification. Faced with the existence of other molecular targets directly or indirectly involved in AD, it opens up a range of possibilities for remediation and therapies [43,60].

#### 2.1.2. The Microtubule-Associated Protein Tau Hypothesis

**Figure 2:**
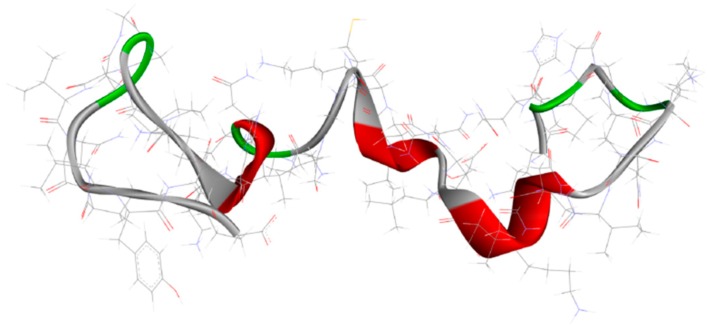
Structure of microtubule-associated protein tau (PDB code: 2MZ7) [64].

An important approach in AD, which tries to interpret and explain the pathology related to the disorder, is shrouded in the tau hypothesis. In this context, NFTs and helically twisted filaments of hyperphosphorylated tau are crucial pathogenetic features in AD. The previously approached β-amyloid hypothesis was proposed in 1991, and it is worth highlighting that the NFT generation is preceded by Aβ deposits [61]. Toxic aggregation and senile plaques formation are related to the unbalance regarding the generation and removal of Aβ from the brain. This frame leads to the hyperphosphorylation process of tau protein, causing a destabilization of the cytoskeleton and larger degeneration of nerve cells. Tau protein (Figure 2) is very important in the sense of stabilizing cytoskeletal microtubules [62] and based on these data, tau becomes a significant biological target for the development of novel effective therapies [63]. These therapies are mostly based on the inhibition of tau phosphorylation, along with microtubule stabilization and prevention of tau oligomerization [47].

In AD, tau phosphorylation is crucial to its function, but in extreme cases of hyperphosphorylated tau, the protein no longer binds to microtubules, but on the contrary, a process of aggregating into paired helical filaments occurs [65]. The outcome is a destabilization of microtubules and disruption of axonal transport, leading to neuronal injury and cell death. The increase in the levels of phosphorylated or total tau in the cerebrospinal fluid (CSF) is a significant indicator of NDDs or injury [66]. Although there is a strong connection between NFT topography and clinical phenotype [67], studies and development of novel therapies having NFTs as molecular targets are not given the same importance as those that target Aβ. The main purposes of these therapies are to decrease, stabilize or prevent hyperphosphorylation or agglomeration of the proteins [47].

### 2.2. Parkinson’s Disease

Parkinson’s disease (PD) is another example of common NDD, reaching around 1% of the population above the age of 60 and about 4% above 85 years old [68]. Among the characteristic symptoms of the illness, it highlights the cognitive impairment generated, being a quite important non-motor aspect of PD, significantly affecting the life quality [69]. PD is characterized by bradykinesia, i.e., a condition where people find difficulties to move their body quickly. In addition, it can be cited a combination of symptoms, such as rigidity, resting tremor, postural instability, along with a range of non-motor symptoms, such as sleep disturbance, constipation, dysarthria, dysphonia, dysphagia, sialorrhoea, urinary incontinence and even constant sleepiness with slight delirium [70,71]. Similar to other NDDs, PD is clinically heterogeneous, presenting variations in some disease-related aspects, for instance, the onset and progression of the disorder. An important fact referring to PD is that the progressive loss of dopamine-containing neurons in the *substantia nigra pars compacta* results in decreased levels of dopamine in the *striatum* [72,73].

Insoluble protein inclusions in neurons, termed Lewy bodies, mainly consisting of aggregated α-Synuclein (αSyn), are the main neuropathological hallmark of PD [74]. Lewy bodies and protein deposits are present in diverse brain regions, spreading with disease progression [75,76]. The exact biological mechanism leading to αSyn aggregation and neuronal loss remains unknown. Currently, only the symptoms of PD are treated with dopamine-replacement therapy, and in some cases, deep brain stimulation [9]. Although there are large investments in the search for neuroprotective compounds for PD, no convincing effects in clinical trials have emerged so far [9]. It is observed that cognitive deterioration in PD people takes place due to the dysmetabolism of both amyloid protein, α-Synuclein and cholinergic dysfunction [77]. Some investigations have shown that a long time occupational exposure to certain chemicals, such as pesticides and heavy metals, is associated with an elevated risk of developing PD [78,79].

### 2.3. Other Known NDDs

Besides Alzheimer’s disease, other NDDs have been associated with misfolding protein aggregation into fibrils that are not completely able to perform their neuronal function.

Amyotrophic lateral sclerosis is a fatal motor neuron disorder characterized by progressive loss of the upper and lower motor neurons at the spinal or bulbar level [80]. It affects about 1–3 per $10^{6}$ individuals. ALS classification is based on the firstly affected area: limb onset with loss of motor capability in arms and legs; or bulbar onset associated with loss of motor neurons that enervate facial and throat muscles. It leads to difficulties in chewing, swallowing, or speaking. The progressive nature of the disease spreads in both instances to all motor neuron populations, although it is slower in limb versus bulbar onset. Both types of ALS patients, however, ultimately succumb to respiratory failure from the loss of diaphragm function and intercostal muscle enervation [12].

Huntington’s disease is a neurodegenerative disorder that has manifestations as chorea, behavioural and psychiatric symptoms and dementia. It is caused by a CAG triplet repeat expansion in the huntingtin gene, which encodes an expanded polyglutamine stretch in the huntingtin protein. The average CAG tract length in the general population is 16 to 20 repeats. In HD, the CAG tract is expanded to 36 repeats or greater. Its clinical diagnosis is based on the development of chorea. It is often observed together with movement abnormalities like dystonia, bradykinesia, and motor incoordination. There are other characteristic behavioural or psychiatric features, such as dementia, personality changes, poor attention, cognitive rigidity, and irritability [81].

Frontotemporal dementia is a disorder of language, cognition and behaviour that affects older segments of society, characterised clinically by progressive behavioural changes and frontal executive deficits and/or selective language difficulties. Some of its most prominent features are progressive aphasia and bizarre affect with personality changes. On average, FTD occurs in patients about a decade earlier than the onset of AD. There are reported cases beginning as early as 21 years old and as late as 80 years old. Apparently, the risk of FTD does not increase with age. Instead, it was found a normal Poisson-like distribution of ages at diagnosis in FTD, with onset arrayed around a mean age of about 62 years. This suggests an underlying pathophysiology in FTD that is less tightly governed by age and differs fundamentally from a condition like AD where the risk of the disease accumulates with age [82,83].

In dementia with Lewy bodies, the defining pathological characteristic is the formation of abnormal protein inclusions called Lewy bodies (LBs) in the cerebral cortex, in brain stem nuclei and parts of the basal forebrain cholinergic system. These inclusion bodies are found in the cytoplasm of cells of a wide variety of subcortical nuclei, including monoaminergic neurons. They are more likely to occur in cortical neurons in patients with PD when the patients also have dementia. A defining constituent is the presence of fibrillar aggregates of alpha-synuclein, a presynaptic protein involved in vesicle formation. The three core clinical diagnostic features of DLB are: cognitive fluctuation (marked variations in attention and alertness occurring over periods ranging from minutes to weeks); mild and spontaneous Parkinsonism (typically bradykinesia and rigidity); and visual hallucinations, such as imagining seeing a family member or pet. Some supporting diagnostic features for DLB include sensitivity to neuroleptic drugs, frequent falls, and rapid eye movement sleep behaviour disorders [84,85].

The Friedreich’s ataxia is a slowly progressive disorder, characterised by a decreased production of the mitochondrial protein frataxin. This deficiency results in abnormal mitochondrial respiration, increased free-radical production, and intramitochondrial iron accumulation in the heart, liver, dentate nucleus of the cerebellum, and fibroblasts. The disorder has, as characteristics, progressive gait and limb ataxia, dysarthria, cardiomyopathy, diabetes, abnormal proprioception and vibratory sense, and loss of reflexes, with a slowly progressive course that culminates in reliance on hands-on assistance for self-care and wheelchair dependence. The usual onset of symptoms is during adolescence (mean 15.5 ± 8 years) with unsteadiness of gait. About 20% of the patients are younger than 5 years at onset. The average time to lose independent gait is 8 years. Patients usually become wheelchair-bound after a mean disease duration of 11–15 years (range 3 to 44 years). The disease onset before 20 years old and cardiac involvement are associated with faster progression of neurological symptoms. Dysarthria manifests within 10 to 15 years and diabetes within 16 years whereas loss of proprioception takes more than 40 years to develop [86,87].

A prion is a protein able to self-replicate. Prion diseases or transmissible spongiform encephalopathies (TSE) are rapidly progressive neurodegenerative disorders caused by the misfolding of the normal cellular prion protein (PrPc) into the disease-causing prion protein (PrPSc), which is perpetuated through an autocatalytic cycle [88]. Prion proteins (PrP) are present in normal cells, but abnormal forms of them may cause infectious diseases by the misfolding of their form [88,89]. Prion disease affects the nervous system of many mammals, including humans [90]. Indeed, because of the huge similarity of the protein aggregation, some scientists do not discard that other NDDs like AD, ALS and other syndromes are caused (and transmitted) by prion-like proteins [91,92].

## 3. Metal-Based Drugs for NDDs Treatment

The understanding of the different factors related with neurodegeneration processes is of great importance to the design of novel treatment methods and therapies. NDDs involve various pathological conditions, which have in common similar critical metabolic processes, for instance, processes related to protein aggregation and oxidative stress. It is demonstrated that these processes are associated with the involvement of metal ions [93]. Metal ions are clearly fundamental for the performance of a series of significant biological functions within the brain, such as nerve transmission, synthesis/metabolism of neurotransmitters, in addition to oxygen transport [94]. It is quite important to highlight the chelating therapy, which could be a remarkable therapeutic approach, keeping in mind that metals are shown to be molecular targets for the rational design of novel therapeutic agents, aiming the treatment of these disorders [93]. Metal-based drugs have demonstrated to be a new and promising alternative to treat NDDs. Particularly, lithium-based treatment has been linked to neuroprotection against neurodegenerative frames, for instance, those observed in PD, AD, and HD as well as ALS [78]. Lithium treatment has indicated to provide neuroprotection against neurological disturbances, including excitotoxicity, ischemic damage and traumatic brain injury [95,96]. Works have indicated that lithium could be an efficient therapy for mood disorders and neurodegenerative conditions. On the other hand, there are many reports about lithium-induced neurotoxicity. High lithium doses are generally required for inducing neurotoxicity, however, it can also take place at therapeutic dosages as well [78,97]. Several lithium studies in AD, PD, and other NDDs have been carried out and included both in vitro and in vivo investigations.

In addition to the already well-known effects of lithium over NDDs, metals such as platinum, copper, zinc, manganese and ruthenium have shown potential benefits for the treatment of NDDs. More details are given in the next section.

### 3.1. Lithium-Based Treatment

Lithium is considered a first-line drug, commonly applied to the treatment of bipolar depression. This element has presented the potential to regulate glycogen synthase kinase-3 (GSK-3); this is a kinase directly related to the phosphorylation process of tau protein [98]. Preclinical investigations have indicated that GSK-3 activity (considering both the GSK-3α and GSK-3β isoforms) could be inhibited by lithium [98,99]. GSK-3β accounts for the phosphorylation process of the majority of the paired helical filament phosphorylation sites [100]. It is noteworthy that the GSK-3α isoform is quite important in the Aβ generation by interacting with γ-secretase [101]. A significant feature of lithium is that it decreases GSK-3 activity in two ways: a direct competition with Mg^2+^ and an indirect increase of phosphorylation of the inhibitory site on GSK-3 [102,103]. Recent in vitro and in vivo works have demonstrated that lithium decreased the phosphorylation of tau protein at AD-specific sites, blocking accumulation of Aβ in the brain [101,104]. In this context, if GSK-3 is related to tau pathology, it shows potential as being a target for possible therapeutic interventions [105].

Lithium salts have been broadly employed in medicine for decades, assisting in the treatment of psychiatric disorders [106]. It is found in the literature reports on one of the first mechanisms of action regarding lithium, which involves the inhibition of inositol monophosphatase (IMP), causing the depletion of inositol triphosphate (IP3). Lately, this outcome has been demonstrated to upregulate autophagy [107,108,109], thus being significant in the prevention or attenuation of neurodegeneration. It is important to keep in mind that autophagy is an intracellular protein degradation pathway, which results in the clearance of mutant and abnormally processed proteins that could accumulate in neurons [109]. As a matter of fact, autophagy-related mechanisms could bring about benefits to a range of animal templates of NDDs [110].

The pharmacological mechanisms referring to lithium are not fully unveiled, but on the other hand, evidence suggests the direct relation of classic pharmacological targets affecting neurotransmission and signal transduction. According to this datum, it highlights the modulation of cell-surface receptors, the release of second messengers and downstream signaling molecules, and also outcomes on the activity of pertinent regulatory systems, influencing on the release of transcription factors and gene expression [111,112]. With more details, it is shown that the monovalent lithium (Li^+^) competes with bivalent magnesium (Mg^2+^) due to the similar ionic radii of these cations, being 0.60 and 0.65 Å, respectively, making lithium capable of binding to Mg^2+^ substrate sites. With the exposed so far, lithium is then capable of inhibiting diverse enzymes that depend on Mg^2+^ as a cofactor [113,114]. The competition observed between lithium and Mg^2+^ on these substrate sites significantly influences the activity of many enzymes. In addition, it is possible to cite some important lithium targets, for instance, glycogen synthase kinase-3 beta (GSK-3β), inositol monophosphatase (IMP) and Akt/β-arrestin2 (Akt stands for Protein kinase B). Interestingly, the modification of these intracellular pathways through enzymatic inhibition is quite significant in the sense of getting a good comprehension of the pathogenesis of specific neuropsychiatric and neurodegenerative disorders [112].

As said previously, GSK-3 has two isoforms, alpha and beta, each one with particular patterns of distribution and homeostatic roles. It is shown that GSK-3β is found in larger amounts in the brain, being involved in cytoskeletal organization and remodeling [115]. Reciprocally, cerebral GSK-3α is related to neurodevelopment, and it is observed certain relation regarding its inhibition by lithium with disease modification, taking into account the transgenic mouse template of AD [101,116]. Undoubtedly, the inhibition of GSK-3β by lithium is one of its most significant mechanisms of action, making it a promising candidate for disease-modifying drugs in the treatment or prevention of AD [112,117].

**Figure 3:**
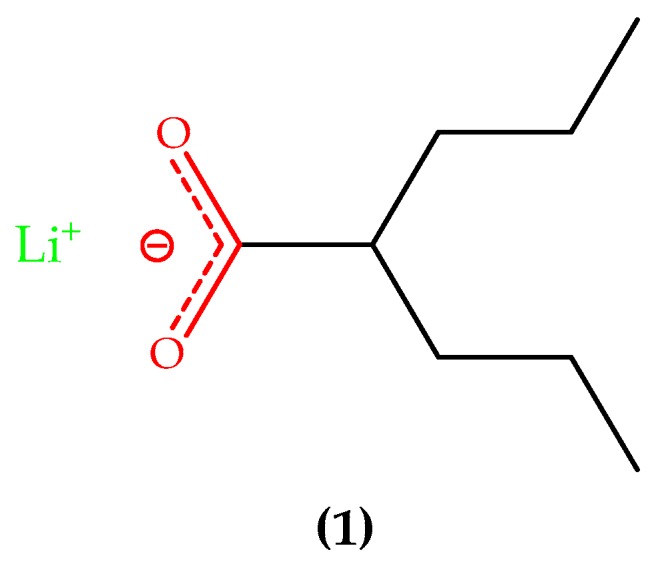
Chemical structure of lithium ion coordinated to valproic acid (**1**) (as valproate).

Recent data in the literature indicate that lithium therapy enhances the mitochondrial respiratory rate, decreases oxidative stress, and protects DNA against damages caused by these oxidative processes, beyond modulating calcium influx in the mitochondria [118,119,120,121,122]. In studies performed with transgenic mice overexpressing GSK-3β, and also considering other animal templates of AD, it was noticed that chronic lithium-based therapies significantly decreased tau phosphorylation [123,124,125]. Furthermore, this kind of treatment decreased Aβ_42_ generation by direct modulation of APP processing and through GSK-3β inhibition [126,127]. It is worth mentioning that the mitigation or reversal of AD-related neuropathology was observed along with a relevant improvement in memory deficits, based on these animal templates [128,129,130]. In addition, lithium could also provide protection for neurons against the neurotoxic effects of Aβ_42_ [131]. Chen and Chuang demonstrated that lithium increases the expression of p53 and Bcl-2, contributing to neuronal survival [132]. Chen et al. [133] indicated that the chronic administration by combining two structurally dissimilar mood stabilizing agents; in this case, lithium plus valproic acid (**1**), see Figure 3, leads to higher levels of Bcl-2 in the cortex, which results in beneficial neuroprotective outcomes. According to Macdonald et al. [134], lithium therapy in AD elderly people presents few side effects and those that were seemingly due to treatment were demonstrated to be mild and reversible.

### 3.2. Inert Complexes Metal Ions

Inspired by the great potential of cisplatin against cancer, many scientists developed their researches in the promising field of inorganic complexes as therapeutic drugs. With NDDs, this trend was not different, and many metallocomplexes were studied to combat those diseases. In the next sections, some inorganic complexes with potential biologic activity are described. They are divided according to their different biological roles.

#### 3.2.1. SODs Mimic Metal-Containing Drugs

**Figure 4:**
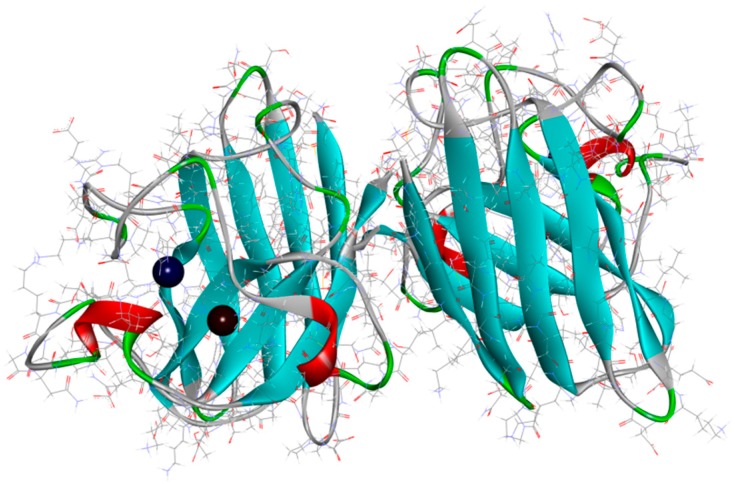
Human superoxide dismutase structure—SOD1 with copper (brown) and zinc (deep blue) ions (PDB code: 2C9V) [137].

Superoxide dismutases (SODs; Figure 4) are metalloenzymes that play the important role of protecting cells of the oxidative stress caused by high concentrations of reactive oxygen species (ROS) like superoxide radical anion (O^2−^). As their name indicates, SODs act as catalysts on the dismutation of O^2−^ to O_2_ and H_2_O_2_. The presence of ROS is normal in cells and is balanced by enzymes like SOD that are able to reduce those harmful species. In some NDDs such as AD and PD, it is documented an excess of free radicals, especially in the brain. In this way, many Cu, Zn and Mn metallodrugs are being tested with the objective of mimic SOD enzymes and act as neuroprotectors due to their antioxidant properties. This idea comes from the fact that SODs contain the cited metal ions in their active site [27,135,136].

**Figure 5:**
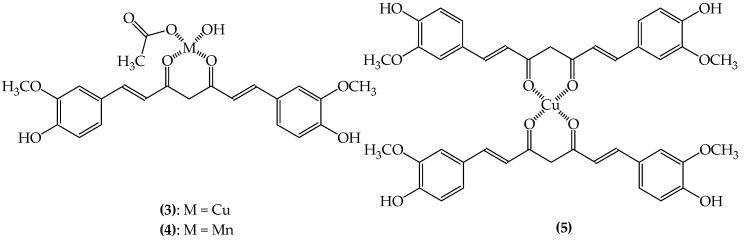
Chemical structures of bioactive metal–curcumin complexes (**3**–**5**).

Metal–curcumin complexes are being studied against oxidative stress in neurons acting as neuroprotective agents. Recent studies reveal that the phenolic hydroxyl groups in curcumin are the main responsible for its antioxidant properties. The intrinsic antioxidant activity of curcumin associated to a favorable non-planar coordination geometry of the complex Cu(Curc)(OAc)(OH) (**3**; Figure 5) yields a powerful superoxide radical anion (O^2−^) reductor catalyst. In addition, studies with similar curcumin complexes but with Mn(II) ions instead of Cu(II)**,** Mn(Curc)(OAc)(H_2_O) (**4**; Figure 5) and Mn(DACurc)_2_ (**5**; Figure 5), showed, beyond the O^2−^ reduction properties, a good NO radical scavenging, becoming a neuroprotector and a potential agent in the treatment of epilepsy and other NDDs induced by oxidative stress [138,139]. Indeed, because of the promising in vitro activity of Mn curcumin complexes, in vivo studies started to be done. Those studies were developed in mice brains and corroborate that Mn curcumin complexes are potent neuroprotective agents and may be used in the treatment of NDDs [140,141].

In another interesting work, Belda et al. [142] synthesized and tested homo and heterobinuclear Cu^2+^ and Zn^2+^ complexes as SOD mimics. The experiments showed high catalytic activity of all tested Cu homobinuclear cyclic hexa-azapyridinocyclophanes, although, for almost all tested ligands, the substitution of one of the Cu^2+^ ions by a Zn^2+^ ion yields a decrease in catalytic dismutase activity. Many other studies need to be done in order to transform those complexes in metallodrugs, but, because of their low IC_50_, some of the studied complexes may act as Cu-SOD mimics and may be also applied as neuroprotective agents [142].

#### 3.2.2. Metal-Based Prion Protein Aggregation Inhibitors

**Figure 6:**
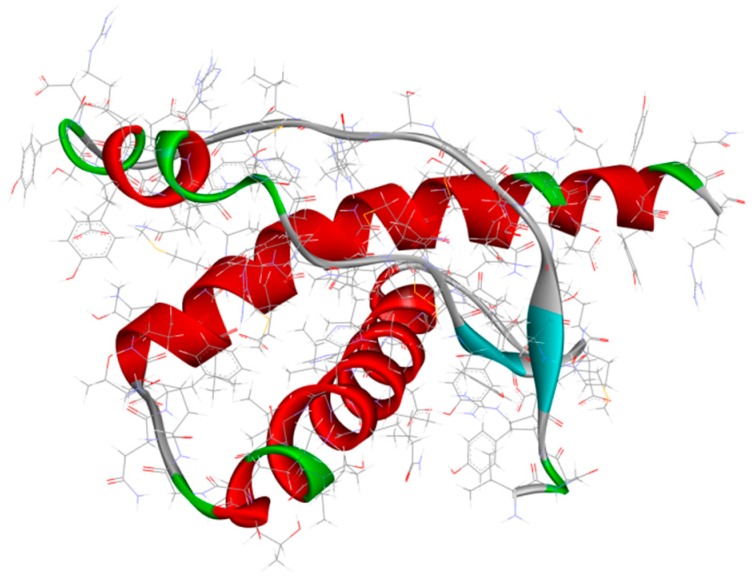
Structure of human prion protein fragment (PDB code: 1QM1) [144].

As said before, a prion (Figure 6) is a protein (without nucleic acid genome) that is able to self-replicate. In this paper, we discuss some complexes that may inhibit PrPs aggregation.

**Figure 7:**
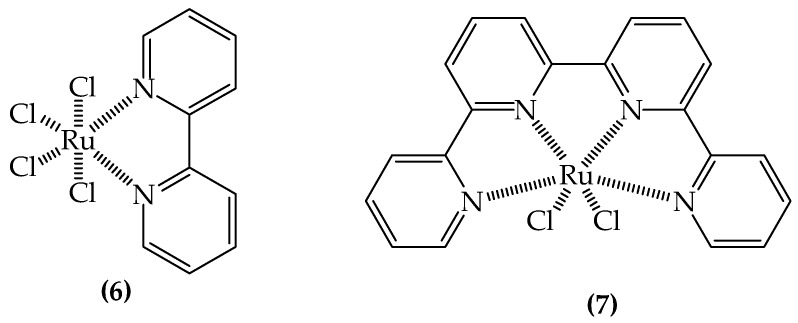
Chemical structure of bioactive Ru complexes (**6**–**7**) [133].

Because of their low cytotoxicity, ruthenium complexes are studied as metallodrugs in the treatment of many diseases. In particular, Wang et al. studied the inhibition of a prion neuropeptide aggregation by six Ru complexes. It was found that all the complexes (like **6** and **7**; Figure 7) could bind to the studied prion known as PrP106–126. The studied aromatic rings ligands were more efficient in the aggregation inhibition [143].

#### 3.2.3. Aβ Aggregation Inhibitors

**Figure 8:**
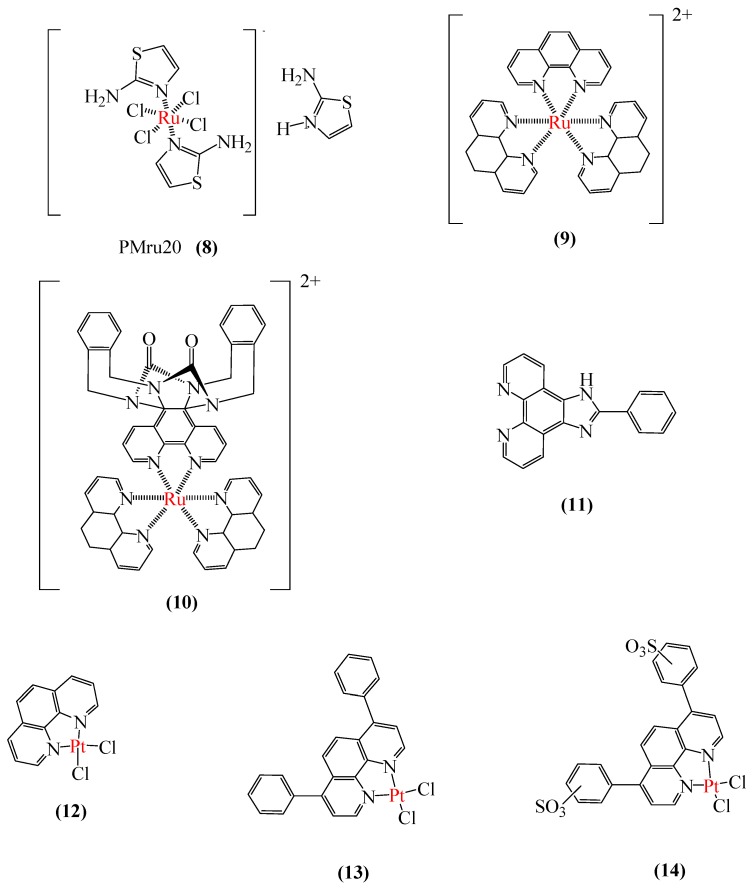
Chemical structures of Aβ-Aggregation Inhibitors, with metal in red color. Molecule **8** are the Ru complex PMRU20; **9** and **10** are Ru(II) polypyridyl complexes; **11** is a phenyl-imidazo-phen ligand, and **12**, **13** and **14** are platinum complexes.

Focusing on AD, Messori et al. [145] studied three Ru(III) complexes as inhibitors of Aβ_42_ aggregation. The authors found out that the complex PMRU20 (**8**) (Figure 8) was very effective in blocking the Aβ_42_ aggregation in comparison with the other two studied complexes. In addition, in vitro experiments indicated low toxicity of the complex, placing the novel complex as a promisor neuroprotective agent. Based on the fact that the acetylcholinesterase (AChE) inhibitors are already a possible treatment for AD [146], Vyas et al. [147] studied Ru(II) polypyridyl complexes (**9**,**10**) (Figure 8) as multi-target drugs: AChE inhibitors and Aβ aggregation blockers. In this very complete work, theoretical and experimental data were analyzed and, for the first time in the literature, it was proposed one drug for those two different purposes.

Another very interesting dual-role metallodrug study was performed recently by Lu et al. [148]. The principal idea of this innovative research was to synthesize and characterize luminescent Ir(III) complexes to act as probes for Aβ_40_ peptide and as inhibitors of its fibrillation. In the mentioned work, 14 iridium complexes were synthesized and tested. Among them, a phenyl-imidazo-phen ligand (**11**) (Figure 8) was the most promising one, inhibiting almost completely the aggregation of Aβ_40_ peptide. In this study, cell viability analysis was performed and it turned out that the complex is able to act as a neuroprotective agent [148].

In a pioneer work and inspired by cisplatin, Barnham et al. [149] studied a set of Pt complexes (**12** to **14**) (Figure 8) on the inhibition of the Aβ peptide and compared the results with cisplatin. In vivo tests were also performed in mice. After this work, many other Pt complexes were tested as therapeutic agents for AD [149,150,151,152]. Despite, just some of those studies showed promising results about the toxicity of the novel complexes. Although permitted Pt drugs are used in different types of treatments (but mainly in chemotherapy), studies reveal that many already approved drugs are responsible for platinum-induced peripheral neurotoxicity. With this indication, it is extremely important to perform rigorous toxicity tests not just on Pt complexes, but on all the others in order to prevent new diseases [153].

In order to enhance the selectivity of Aβ and consecutively prevent side effects, Li et al. [154] introduced a novel class of chiral inhibitors. The work is based on the fact that the α-helix in the 13−23 segment of the Aβ enzyme has a critical role in the fibril formation. Thus, the authors took advantage of the chirality of the l-amino acids and the 3D peptide structure of the α-helix to develop chiral Fe supramolecular complexes. The metallocomplexes are able to enantioselectively inhibit Aβ fibrillation. In addition, experimental tests also showed a superoxide dismutase activity of the compounds [154]. This work opened a new important door to selective drugs in AD.

## 4. Chelating Agents

As previously mentioned, many studies indicate that abnormal metal homeostasis is an important pathogenic factor in many neurodegenerative diseases [155]. The imbalance of metal ion concentrations in the brain could be responsible for damages related to neuronal cell apoptosis [156]. The first kind of metal-promoted damage is the oxidative stress. The aggregation of peptides, like Aβ peptide, with metal ions, such as copper, zinc or iron, promotes the intracellular accumulation of ROS [157]. High concentrations of ROS species promote degradation of many cells and tissues, leading to several pathophysiological conditions, including PD and AD [156]. A secondary consequence of metal imbalance is the protein modification and aggregation, which induces the formation of intra and extracellular Aβ peptide deposits [156]. In addition, it is demonstrated, through in vitro studies, that high concentrations of metal ions, such as Cu(II) and Fe(III), allow these charged species to bind to and increase the fibrillization of the α-synuclein protein [158].

Having in mind all these problems related to the presence of metal ions in the brain, many studies have been developed exploring the potential of chelating agents, which are capable of capturing the redox-active metal [155,159]. In order to be an eligible chelating agent in the treatment of AD, it must present a low molecular weight, chelation selectivity, avoiding depletion of other metal ions, and it must be able to tear off the metal ions of pathogen proteins [160]. Furthermore, suitable chelating agents should be capable of capturing the free redox active metal, besides preventing the ROS production [155].

### 4.1. Multifunctional Agents

The incorporation of chelating properties in a single molecule is the main characteristic of multifunctional compounds (MFCs) [161]. The development of these multitarget-directed ligands (MTDLs) has been one of the main focuses of current research in the search for AD drugs [162].

**Figure 9:**
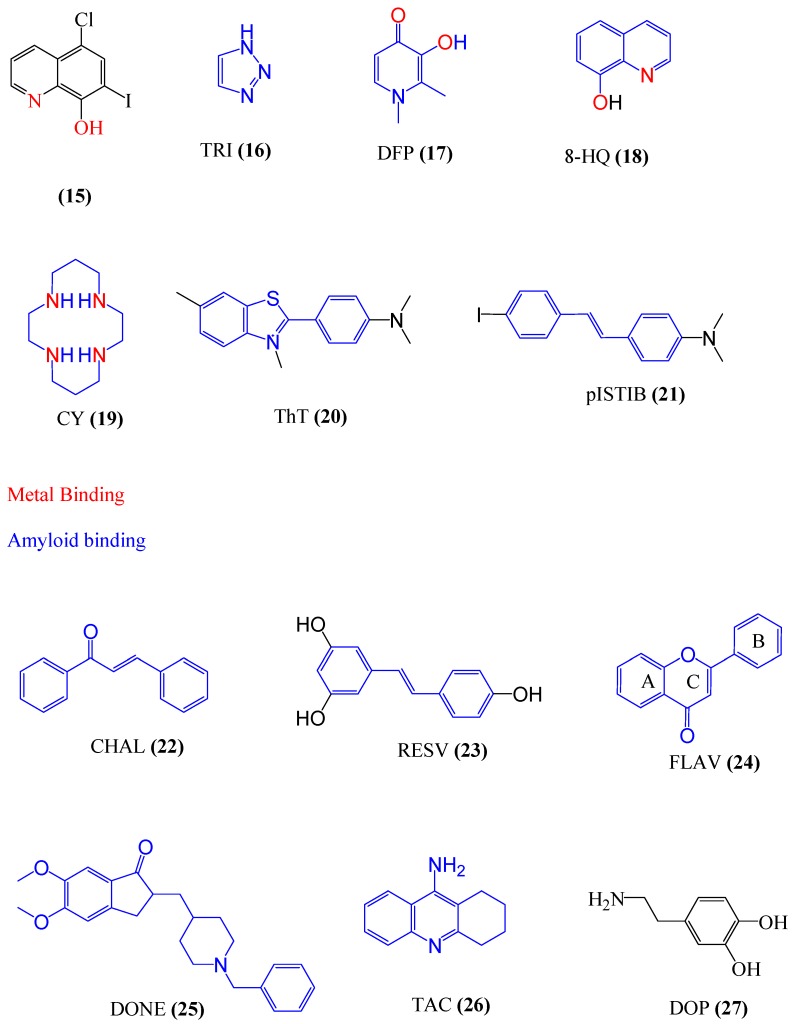
Template molecules for the development of multifunctional compounds. Clioquinol molecule, 5-chloro-7-iodo-quinolin-8-ol (**15**); triazole (TRI), 1,2,3,4-tetrahydroacridin-9-amine (**16**); diferiprone (DFP), 1,2-dimethyl-3-hydroxypyridin-4-on (**17**); 8-hydroxyquinoline (8-HQ) (**18**), cyclam (CY) (**19**), thioflavine T (ThT), 4-(3,6-dimethyl-1,3- benzothiazol-3-ium-2-yl)-N,N-dimethylaniline (**20**); *p*-I-stilbene (pISTIB) (E)-4-iodo-4′-dimethylamino-1,2-diphenylethylene (**21**); chalcone (CHAL), (2E)-1,3-diphenylprop-2-en-1-one (**22**), resveratrol (RESV), 3,5,4′-trihydroxy-trans-stilbene (**23**); flavone (FLAV), 2-phenyl-4H-1-benzopyr-4-one (**24**); donepezil (DONE), 2-[(1-benzylpiperidin-4-yl)methyl]-5,6-dimethoxy-2,3-dihydro-1H-inden-1-one (**25**), tacrine (TAC), 1,2,3,4-tetrahydro-9-acridinamine (**26**) and dopamine (DOP), 4-(2-amino-ethyl)-benzene-1,2-diol (**27**).

The first metal ligand used as a therapeutic agent was clioquinol (CQ) (**15**) (Figure 9), a 8-hydroxyquinoline derivative [163]. However, it was verified that a long-term use of CQ produces several side effects [160]. In this sense, a wide range of molecules has been developed, focusing on possible multitarget drugs for the treatment of AD and other NDDs. Among these drugs, there may be highlighted the molecules derived from various compounds, as triazole (TRI) (**16**) [164,165,166,167,168,169] diferiprone (DFP) (**17**) [170,171,172,173], 8-hydroxyquinoline (8-HQ) (**18**) [163,174,175,176,177,178], cyclam (CY) (**19**) [179,180,181,182,183], thioflavine T (ThT) (**20**) [19,184,185,186,187], *p*-I-stilbene (pISTIB) (**21**) [188,189,190,191,192,193,194,195,196], chalcone (CHAL) (**22**) [167,168], resveratrol (RESV) (**23**) [197,198], flavone (FLAV) (**24**) [199,200], donepezil (DONE) (**25**) [201,202,203,204,205], tacrine (TAC) (**26**) [206,207,208,209,210,211,212,213,214,215,216,217,218,219], dopamine (DOP) (**27**) [220] and peptide-based inhibitors (PEP) [221,222,223], which can be seen in Figure 9. These derived molecules, besides presenting chelating property, can also act on different molecular targets, such as oxidative stress and Aβ, cholinesterases (ChEs), tau and monoamine oxidases (MAOs) enzymes [12]. Some molecules already developed with some of these templates are described below.

**Figure 10:**
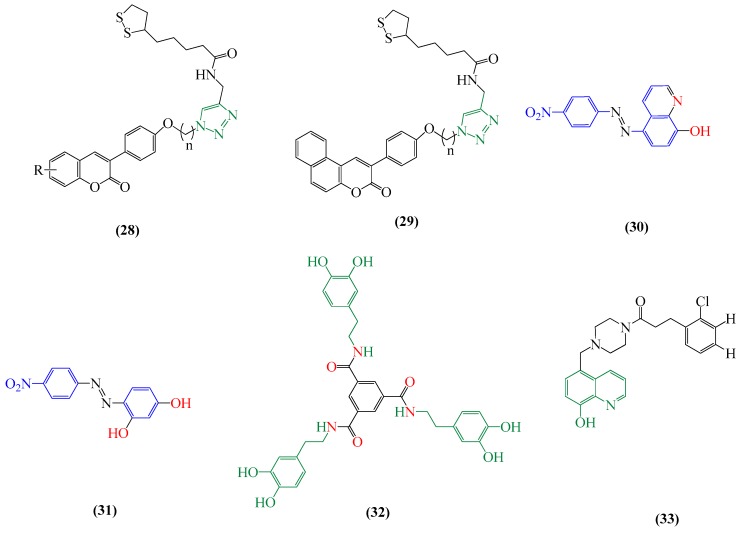
Chemical structures of the multifunctional compounds for ND disease treatment. **15** is the clioquinol molecule, 5-chloro-7-iodo-quinolin-8-ol **28** and **29** are 3-arylcoumarines derivatives, **30** is 5-((4-nitro-phenyl)diazenyl)quinolin-8-ol (HL1), **31** is the 4-((4-nitrophenyl)diazenyl)benzene-1,3-diol (HL2), **32** is the tris(dopamine) derivative benzene-1,3,5-tricarboxylic acid tris-{[2-(3,4-dihydroxy-phenyl)-ethyl]-amide} and **33** is an 8-hydroxyquinoline derivative 3-(2-chloro-phenyl)-1-[4-(8-hydroxy-quinolin-5-ylmethyl)-piperazin-1-yl]-propan-1-one.

Baleh and collaborators [157] developed a novel series of TRI derivatives (**28**,**29**) (Figure 10), which act as MTDL and also as neuroprotective agents. Most of the compounds showed neuroprotection effects increasing the cell viability in the presence of H_2_O_2_, anti-cholinesterase activity (varying the effect according to the length of carbon spacer and being a four-carbon spacer the best inhibitor), good antioxidant activity and metal chelating properties. Based on the idea of developing metal chelators from functionalized small molecules with amyloid recognition units, the 8-HQ, pISTIB and RESV structures were used to develop the azo dyes-based compounds **30** and **31** (Figure 10). Compounds 30 and 31 are able to bind on Cu^2+^ ions and control amyloid formation [151]. However, cell toxicity studies revealed that azo-dyes compounds, although non-toxic, may present significant toxicity when a nitro group substitution occurs.

Regarding the iron-chelating agents, one effective compound is tris(DOP) derivative L1H6 (**32**) (see Figure 10) [215]. This molecule has a favorable geometric arrangement when coordinated to Fe(III) ion. The accumulation of iron can induce many neurodegenerative disorders, including PD, and the high affinity and selectivity of compound **32** allow it to be applied in situations where the concentrations of other essential ions, such as Zn(II) and Mg(II), should be maintained [215]. In addition, these molecules have great potential to act as antioxidant agents and show relatively low cytotoxicity.

The ability of 8-HQ derivatives to act as MTDLs was tested by Yang and collaborators [174]. The compounds substituted with Cl and H (**33**), as shown in Figure 7, reveal great potential in exerting metal-chelating activity, among other properties, such as antioxidant effect and inhibition of Aβ aggregation. Tacrine-hydroxyphenylbenzimidazole hybrids also show moderate metal chelating ability, as well as inhibitory activity against AChE and capacity for recovering cholinergic neurons [18].

Metal binding peptides, such as methanobactin (Mb) from *Methylosinus trichosporium* OB3b, are also a drug alternative [224]. The Mbs are peptides secreted by methanotrophs in response to low concentrations of copper ions in their environment. These peptides are catalytic redox enzymes, which have a preference for copper ions, reducing Cu(II) to Cu(I). The potential of this class of peptides as chelating agents for the treatment of NDDs, related to alterations of copper ions in the organism, has been studied continuously. Selectivity tests were done employing Ag(I), Pb(II), Co(II), Fe(II), Mn(II), Ni(II) and Zn(II). As results, the authors found that the selectivity of Mb peptide, at pH 6.5, follows the order Ag(I) ≈ Cu(I) > Ni(II) ≈ Zn(II) > Co(II) >> Mn(II) ≈ Pb(II) > Fe(II). On the other hand in a pH range from 7.5 to 10.4, the selectivity order is changed by: Ag(I) > Cu(I) > Ni(II) > Co(II) > Zn(II) > Mn(II) ≈ Pb(II) > Fe(II). The results are an expansion of previous works and were able to determine the reaction products of Mb for selected metal ions, which can be useful in the treatment of NDDs.

Many other compounds have been studied with this same chelating purpose, such as propargylamine-modified pyrimidinylthiourea derivatives [159], iminochromene-2H-carboxamide derivatives [225] hydroxy-substituted trans-cinnamoyl derivatives [226], CQ derivatives [160], phenanthroline derivatives [227], pyrrolidine dithiocarbamate [228], macrocyclic polyamine [160,229] among many others [12]. As the cause of these diseases is multiple, the incorporation of many functions in a single molecule is a promising strategy to improve NDDs treatment [230].

### 4.2. Drug Repositioning for Chelating Agents

**Figure 11:**
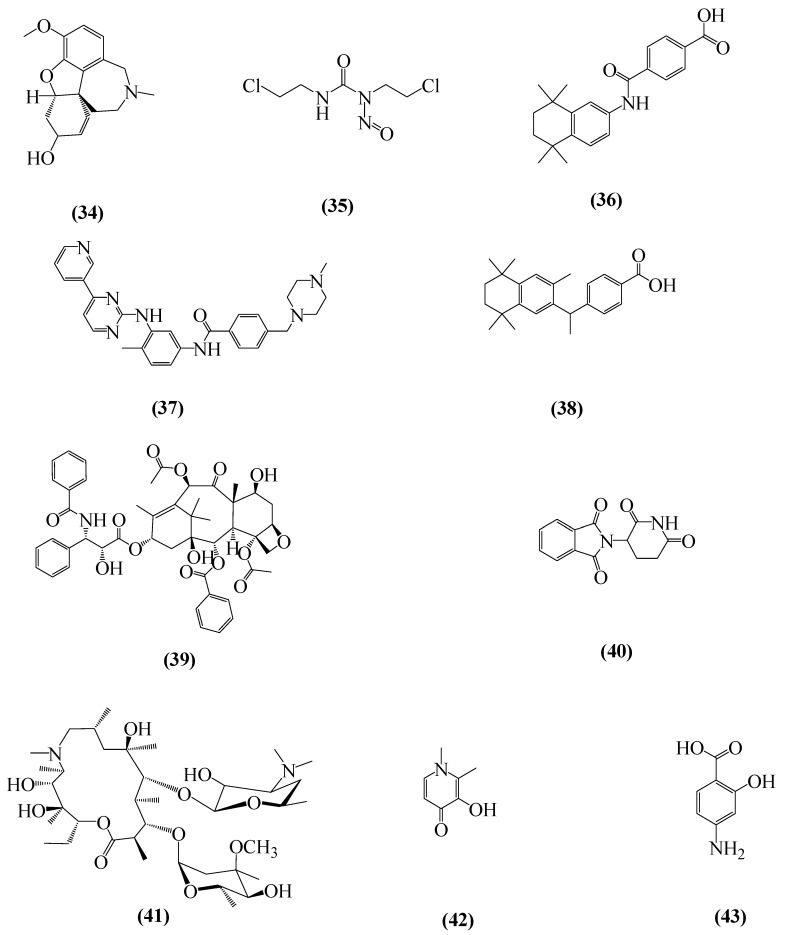
Chemical structures of commercialized drugs proposed for NDD treatment: galantamine, (4aS,6R,8aS)-5,6,9,10,11,12-Hexahydro-3-methoxy-11-methyl-4aH-[1]benzofuro[3a,3,2-ef][2]benzazepin-6-ol (**34**); casmustine (**35**), 1,3-Bis(2-chloroethyl)-1-nitrosourea; tamibarotene (**36**), 4-[(1,1,4,4-tetramethyltetralin-6-yl)carbamoyl]benzoic acid; imatinib (**37**), 4-[(4-methylpiperazin-1-yl)methyl]-N-[4-methyl-3-[(4-pyridin-3-ylpyrimidin-2-yl)amino]phenyl]benzamide; bexarotene (**38**), 4-[1-(5,6,7,8-tetrahydro-3,5,5,8,8-pentamethyl-2-naphthalenyl)ethenyl]benzoic acid; paclitaxel (**39**), (2α,4α,5β,7β,10β,13α)-4,10-bis(acetyloxy)-13-{[(2R,3S)-3-(benzoylamino)-2-hydroxy-3-phenylpropanoyl]oxy}-1,7-dihydroxy-9-oxo-5,20-epoxytax-11-en-2-yl benzoate; thalidomide (**40**), 2-(2,6-dioxopiperidin-3-yl)-2,3-dihydro-1H-isoindole-1,3-dione; azythromicin (**41**), (2*R*,3S,4*R*,5*R*,8*R*,10*R*,11*R*,12S,13S,14*R*)-2-ethyl-3,4,10-trihydroxy-3,5,6,8,10,12,14-heptamethyl-15-oxo- 11-{[3,4,6-trideoxy-3-(dimethylamino)-β-d-xylo-hexopyranosyl]oxy}-1-oxa-6-azacyclopentadec-13-yl 2,6-dideoxy-3C-methyl-3-*O*-methyl-α-l-ribo-hexopyranoside; deferiprone (**42**), 3-hydroxy-1,2-dimethylpyridin-4(1H)-1 and 4-aminosalicylic acid (**43**), 4-Amino-2-hydroxybenzoic acid.

An interesting strategy for the development of new NDDs therapies is drug repositioning, also known as drug reprofiling or drug repurposing [231]. This methodology consists in the identification of new therapeutic applications for existing drugs [232]. The great advantage of this method is the low investment in time and cost [233] since the data about pharmacokinetic, toxicology and safety are already existent [232]. Many commercialized drugs have been repositioned for AD treatment, acting through different mechanisms of action. Among them are the galantamine (**34**) [234], used in AD, carmustine (**35**) [235], tamibarotene (**36**) [236], imatinib (**37**) [237], bexarotene (**38**) [238], paclitaxel (**39**) [239], thalidomide (**40**) [240] and azithromycin (**41**) [241], all represented in Figure 11.

Preclinical tests strongly support evidence of deferiprone (**42**) as neuroprotector [242,243]. Traditionally used in the treatment of thalassemia major, deferiprone is a chelating agent for intracellular iron that has been proposed for PD treatment [244]. Due to its physicochemical characteristics, such as a favorable partition coefficient, low molecular weight and neutral charge, deferiprone is able to cross the BBB [245]. Considering that iron is a key factor in the progression of PD, the removal of excess cerebral iron may be a useful strategy in its treatment [246]. Deferiprone can redistribute the excess of intracellular iron to the extracellular apotransferrin, which is important in avoiding systemic iron losses, different from other chelating agents [247]. The non-steroidal anti-inflammatory drug 4-aminosalicylic acid (**43**) also has neuroprotective effects and prevents Mg^2+^ accumulation [248].

As already mentioned, there is an advantage to employ known drugs in new treatments, since these drugs have a longer study time, and many parameters are better established. With respect to pharmacogenetics, for Donepezil and Galantamine, for example, 80 allelic variants have been described due to genetic polymorphisms in CYP2D6 gene [249]. These studies have been performed considering the fact that the Cytochrome P450 (CYP) 2D6 enzyme is the major responsible for the metabolism of this drugs and that functional polymorphisms in CYP2D6 gene can affect. Resistance to Imatinib treatment also has been described, and genetic variation the activity of CP450, and consequently affect the drugs metabolism [250,251,252,253,254,255] ns in influx transporter gene SLC22A1 [255] as well as ABCB1 [256] are associated with this resistance. For the last, CAC haplotype was associated with a high level of resistance, once the 1236C-2677A-3435C haplotype have been found only in resistant patients. For Paclitaxel, the resistance is linked to high expressions of gene encoding P-glycoprotein (PGP). In addition, the use of this drug increases the expression of the MDR1 gene by altering cellular mechanisms [257,258]. In relation to toxicity, the expression of the ABCB1 gene (also known as MDR1, encoding the P-glycoprotein) have studied, and an overexpression of MDR1 is the strongest predictive biomarker of taxanes (Paclitaxel and Docetaxel) resistance in general [257]. Pharmacogenetic studies of adverse drug response to Deferiprone found that the UGT1A6 2 Arg184Ser polymorphism of the UGT1A6 gene may interfere in adverse drug reactions (ADR), but not in drug resistance [259].

As can be seen, there are many molecules already developed that can be used as chelating agents. Besides the ability of chelating metal ions, it seems appropriate to consider other perspectives, such as side effects, toxicity, adverse effects and many other factors. It is also important to consider that a powerful chelating agent can remove more metal ions from the body than necessary, which may compromise cellular functions [251]. In this sense, a new class of compounds has been studied, called metal protein attenuating Compounds (MPACs).

### 4.3. Metal Protein Attenuating Compounds (MPACs)

Metal protein attenuating compounds (MPACs) are a different class of chelating agents which moderate affinity for metal ions. As previously mentioned, copper and zinc ions are responsible for mediating the Aβ aggregation and toxicity, as well as the production of neurotoxic hydrogen peroxide. In this way, the removal of these metal ions promotes the solubilization of Aβ [260]. MPACs compete with the target protein for metal ions, correcting abnormal concentrations of these metals, which means that their affinity for metal ions is reduced, compared to traditional chelating agents [261]. This concept should not be exchanged by the concept of “chelating agents” mentioned above. Chelating metals are responsible to remove bulk metals, as in the case of Wilson’s disease, which involves abnormal concentrations of copper ions [261]. The MPACs are capable of crossing the BBB and have subtle effects on metal homeostasis, decreasing oligomerization of Aβ by inhibiting Zn^2+^ and Cu^2+^ ions [17]. This compounds may have the characteristic of both solubilizing and rescinding the oxidation and toxicity of Aβ peptide mediated by metal ions [260].

**Figure 12:**
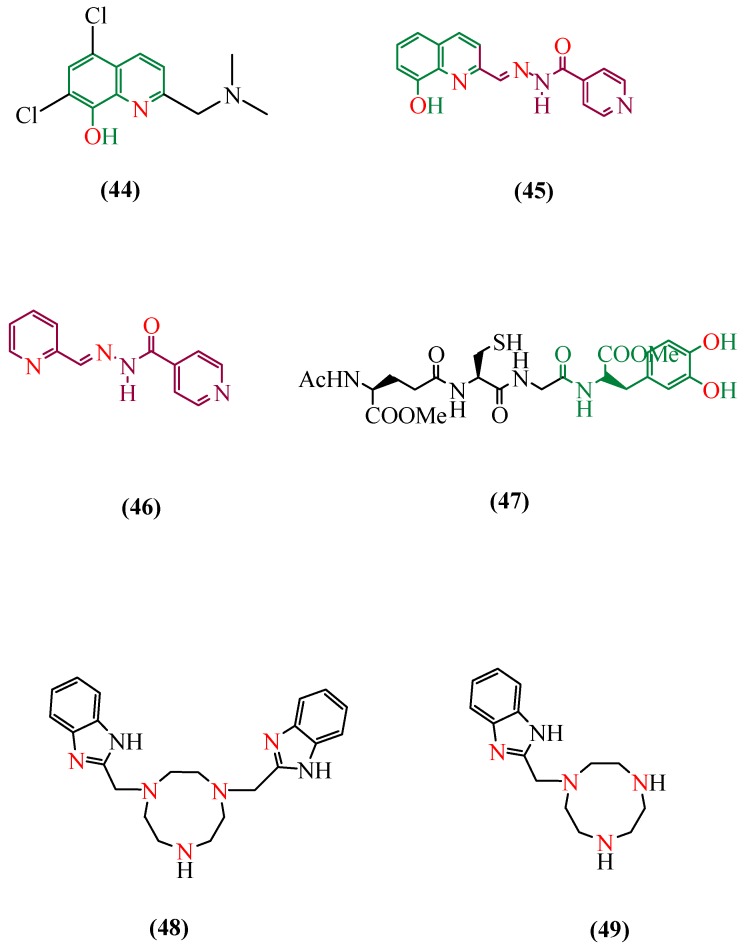
Chemical structures of metal protein attenuating compounds PBT2, (**44**), 5,7-Dichloro-2-dimethylaminomethyl-quinolin-8-ol; INHQ (**45**), 8-hydroxyquinoline-2-carboxaldehyde isonicotinoyl hydrazone; HPCH (**46**), pyridine-2-carboxaldehyde isonicotinoyl hydrazone; GSH-LD (**47**), 2-Acetylamino-4-[1-({[2-(3,4-dihydroxy-phenyl)-1-methoxycarbonyl-ethylcarbamoyl]-methyl}-carbamoyl)-2-mercapto-ethylcarbamoyl]-butyric acid methyl ester; 1-BYT (**48**), 1-(benzimidazole-2-ylmethyl)-1,4,7-triazacyclononane and 1,4-BYT (**49**), 1,4-bis(benzimidazole-2-ylmethyl)-1,4,7-triazacyclonone.

The first compound used as MPAC was the previously mentioned CQ (15) [260]. This molecule is able to restore metal homeostasis, to reduce levels of Aβ peptides, among other positive effects [262]. Initially used as an oral antibiotic [263], CQ was withdrawn from the market after presenting side effects related to neurological effects [264]. Clioquinol seems to favor the entrance of copper and zinc ions into cells, triggering activation of metalloproteases and degrading Aβ [264,265]. The ability to cross the BBB and the approval by the U.S. agency FDA (Food and Drug Administration) have also motivated its use [266]. A pilot phase 2 of a clinical trial was conducted by Ritchie and collaborators and, over 36 months, 36 patients participated in the test [267]. CQ treatment showed a decrease in cognitive deterioration and a reduction in Aβ levels, maintaining copper levels in plasma. Despite the promising results, large scale synthesis limitation and side effects [268], such as the induction of myelinopathies [269], led to the discontinuation of the studies. The second generation of 8-hydroxyquinoline derivatives was developed. One of these derivatives, PTB2 (44) (Figure 12), has shown to be more promising than CQ, with fewer side effects. PTB2 has a higher solubility, easier chemical synthesis, and also increases BBB permeability [268]. However, despite presenting good safety and tolerability on patients with mild AD, a phase II of the clinical trial did not show a considerable reduction of Aβ concentrations [230,270,271]. Nowadays, PBT2 is in clinical development for the treatment of HD [272].

In order to improve the metal affinity, among other features, the 8-HQ derivative with an arylhydrazone moiety, INHHQ (**45**) [273] (Figure 12) was developed. INHHQ shows in vitro MPAC activity, being nontoxic for male Wistar rats and able to interact with Cu(II) and Zn(II) from Aβ. Besides that, it is able to cross the BBB, disrupt anomalous Cu-α-Syn interactions, and also inhibits oligomerization of this enzyme, which is an advantage in PD treatment [272]. In order to investigate the role of arylhydrazone moiety in MPAC activity, the compound HPCIH (**46**) (Figure 12) [274] was tested, and it also showed competition with Aβ for Zn^2+^ ions. Peptidic ligands can also act as a MPAC compound. GSH-LD (**47**) (Figure 12) is an example. Together with L-Dopa, the GSH, a peptide known for its pleiotropic action in NDDs, was employed for the development of the GSH-LD molecule. GSH-LD is able to selectively remove the excess of Cu^2+^, and partially Zn^2+^ excess from Aβ peptide. Biological tests revealed that the compound could also counteract oxidative stress.

Aiming to potentialize the MPACs’ effects, SOD-mimics compounds were tested by Ji and Zang [275]. Considering that SOD-mimics are metal chelating agents, the authors evaluated four compounds with possible chelating activity close to that of CQ. Based on theoretical calculations [276,277], it was found that the compounds 1-BYT (**48**) and 1,4-BYT (**49**) exhibited metal-chelating ability similar to clioquinol, having the advantage of being a SOD-mimic compound. The imidazole group found in both compounds have many sites in which modifications may be made, aiming to reach other interesting properties such as prevention of Aβ aggregation. This is a possible pathway for the development of new, more specific and potent MPACs for the treatment of NDDs [265].

## 5. Conclusions

The present review provides a description of some of the alternatives that are currently being developed for the treatment of neurodegenerative diseases, regarding metallic ions, metallodrugs and also chelating agents. It is known that metal ions are fundamental to organisms and perform many significant biologic functions of great importance. It is also known that abnormal metal homeostasis is a relevant pathogenic factor for NDDs. Having in mind that only some symptomatic treatments are currently available, the implantation of new treatments is still a great challenge and can improve the quality of life of patients with these diseases and even their cure. Based on the fact that many compounds are already developed and present great potential for implantation in the remediation process. Nonetheless, many of these compounds also have great potential to be used in NDDs. However, in practical terms, a great amount of work needs to be done to optimize the conditions of use and make the introduction of new compounds feasible in the market. Moreover, there should be an improvement in their pharmacodynamics and pharmacokinetics, improving not only the permeability of some of these compounds in the BBB, but also its mode of action and specificity, among other aspects.

## Abbreviations

8-HQ	8-Hydroxyquinoline
Aß	Amyloid-ß
AD	Alzheimer’s disease
ALS	Amyotrophic lateral sclerosis
APP	Amyloid precursor protein
BBB	Blood-brain barrier
CHAL	Chalcone
ChEs	Cholinesterases
CNS	Central nervous system
CQ	Clioquinol
CSF	Cerebrospinal fluid
CY	Cyclam
DFP	Diferiprone
DLB	Dementia with Lewy bodies
DONE	Donepezil
DOP	Dopamine
FLAV	Flavone
FNHK	
FRDA	Friedreich’s ataxia
FTD	Frontotemporal dementia
GSK-3	Synthase kinase-3
HD	Huntington’s disease
IMP	Inositol monophosphatase
IP3	Inositol triphosphate
MAOs	Monoamine Oxidases
Mb	Methanobactin
MFCs	Multifunctional compounds
MPACs	Metal Protein Attenuating Compounds
MTDLs	Multitarget-directed ligands
NDDs	Neurodegenerative diseases
NFTs	Neurofibrillary tangles
PD	Parkinson’s disease
PDB	Protein Data Bank
pISTIB	*p*-I-stilbene
P-Tau	Hyperphosphorylated tau
RESV	Resveratrol
ROS	Reactive oxygen species
SOD	Superoxide dismutases
TAC	Tacrine
ThT	Thioflavine T
TRI	Triazole
UHK	
